# A Novel Nonsense Mutation in the *DMP1* Gene Identified by a Genome-Wide Association Study Is Responsible for Inherited Rickets in Corriedale Sheep

**DOI:** 10.1371/journal.pone.0021739

**Published:** 2011-07-01

**Authors:** Xia Zhao, Keren E. Dittmer, Hugh T. Blair, Keith G. Thompson, Max F. Rothschild, Dorian J. Garrick

**Affiliations:** 1 Department of Animal Science and Center for Integrated Animal Genomics, Iowa State University, Ames, Iowa, United States of America; 2 Institute of Veterinary, Animal and Biomedical Sciences, Massey University, Palmerston North, New Zealand; Arizona State University, United States of America

## Abstract

Inherited rickets of Corriedale sheep is characterized by decreased growth rate, thoracic lordosis and angular limb deformities. Previous outcross and backcross studies implicate inheritance as a simple autosomal recessive disorder. A genome wide association study was conducted using the Illumina OvineSNP50 BeadChip on 20 related sheep comprising 17 affected and 3 carriers. A homozygous region of 125 consecutive single-nucleotide polymorphism (SNP) loci was identified in all affected sheep, covering a region of 6 Mb on ovine chromosome 6. Among 35 candidate genes in this region, the dentin matrix protein 1 gene (*DMP1*) was sequenced to reveal a nonsense mutation 250C/T on exon 6. This mutation introduced a stop codon (R145X) and could truncate C-terminal amino acids. Genotyping by PCR-RFLP for this mutation showed all 17 affected sheep were “T T” genotypes; the 3 carriers were “C T”; 24 phenotypically normal related sheep were either “C T” or “C C”; and 46 unrelated normal control sheep from other breeds were all “C C”. The other SNPs in *DMP1* were not concordant with the disease and can all be ruled out as candidates. Previous research has shown that mutations in the *DMP1* gene are responsible for autosomal recessive hypophosphatemic rickets in humans. Dmp1_knockout mice exhibit rickets phenotypes. We believe the R145X mutation to be responsible for the inherited rickets found in Corriedale sheep. A simple diagnostic test can be designed to identify carriers with the defective “T” allele. Affected sheep could be used as animal models for this form of human rickets, and for further investigation of the role of DMP1 in phosphate homeostasis.

## Introduction

Rickets is a metabolic bone disease in humans and animals with most cases caused by a nutritional deficiency of either vitamin D or phosphorus. This disease leads to softening and weakening of bone caused by defective mineralization of cartilage at sites of endochondral ossification and potentially causes fractures and limb deformities [1], [2], [3]. Rickets may also result from genetic mutations that disrupt genes whose functions are critical for normal bone metabolism. Five types of rickets have been described in humans with common clinical characteristics of renal phosphate wasting, including X-linked hypophosphatemic rickets (XLH), autosomal dominant hypophosphatemic rickets (ADHR), autosomal recessive hypophosphatemic rickets (ARHR) type 1 and 2, and hereditary hypophosphataemic rickets with hypercalciuria (HHRH). A large number of different mutations comprising single base pair deletions, nonsense and missense mutations have been identified in *PHEX* (phosphate regulation gene with homologies to endopeptidases on the X chromosome) as being responsible for XLH [4], [5], [6]. ADHR is caused by gain of function mutation leading to increased activity of fibroblast growth factor 23 encoded by gene *FGF23* [7], and mutations in the dentin matrix protein 1 gene (*DMP1*) and ecto-nucleotide pyrophosphatase/phosphodiesterase 1 (*ENPP1*) have been identified in autosomal recessive hypophosphataemic rickets type 1 and 2, respectively [8], [9], [10]. The mutation for HHRH occurs in the gene for the NaP_i_-IIc protein (or *SLC34A3*, human chromosome 9q34), a renal Na-P co-transporter [11]. Two other recessively inherited forms of rickets in humans are due to the disruptions of either the synthesis or metabolism of vitamin D. Vitamin D-dependent rickets type I (VDDR-I) is caused by mutations in the *CYP27B1* gene, which encodes vitamin D 1-alpha-hydroxylase [12]. Loss of function mutations in the vitamin D receptor gene (VDR) are the genetic basis for vitamin D-dependent rickets type II (VDDR-II), which is also called hereditary vitamin D - resistant rickets (HVDRR) [13], [14].

**Figure 1:**
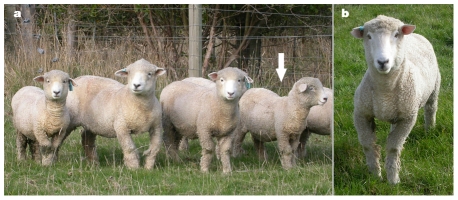
Corriedale sheep with rickets. (**a**) 1½-year-old Corriedale sheep with inherited rickets showing angular limb deformities of the forelimbs and lordosis of the spine in the mid-thoracic region (white arrow). (**b**) 1½-year-old Corriedale sheep with inherited rickets showing combined varus and valgus or “windswept” deformity of the forelimbs.

Inherited forms of rickets have been uncovered frequently in humans but were rare in domestic animals. Recently, an inherited form of rickets has been described in purebred Corriedale sheep from a commercial flock in New Zealand, with an incidence of up to 20 lambs out of 1,600 over a 2-year period. Affected sheep were characterized by decreased growth rate, thoracic lordosis and angular limb deformities (**Figure 1**) [15], with low serum calcium and phosphate concentrations and normal 25 hydroxyvitamin D and 1,25 dihydroxyvitamin D_3_ concentrations [16]. Embryo transfer and backcross breeding trials have determined that this disease is likely a simple autosomal recessive disorder [16].

Large animal models of human genetic diseases have received extensive clinical attention due to their advantages over rodent models, including greater physiological similarity to human patients, a longer life span and larger body size. Sheep have been used as an animal model for gene therapy (GT) of human diseases, such as post-traumatic osteoarthritis [17] and steroid-induced ocular hypertension [18]. The convenience of performing in utero GT prior to disease onset will help to achieve long-term expression of the exogenous gene without modifying the germ line of the recipient [19]. Based on the hypothesis that inherited rickets-affected Corriedale sheep could be a potential model for human rickets; and a genetic marker could help sheep breeders to avoid at-risk matings, we performed a genome-wide association study by genotyping 54,241 evenly distributed single nucleotide polymorphisms (SNPs) for 17 affected Corriedale sheep and 3 carriers using the Illumina Ovine SNP50 BeadChip. It has been shown that the analysis of genome-wide high-density SNPs is an effective strategy to map and identify causal mutations for recessive diseases [20].

## Results

### Homozygosity Mapping

**Figure 2:**
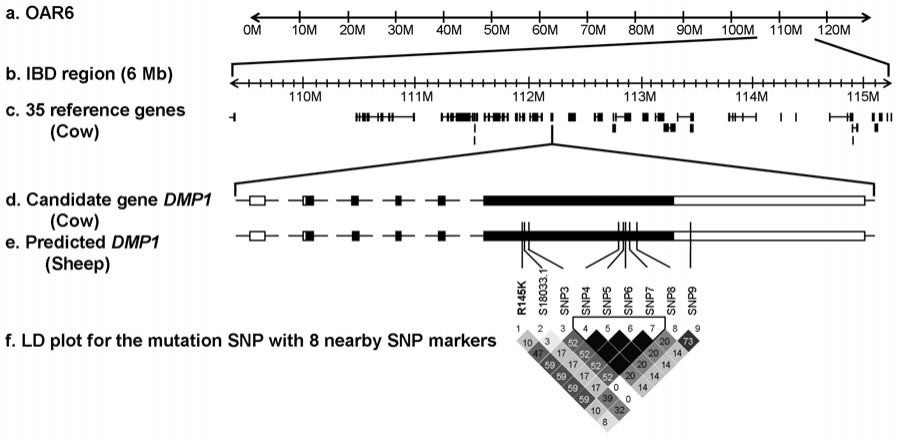
Work flow chart for discovering the mutation locus of inherited rickets in Corriedale sheep. (**a**) The sheep chromosome 6 (OAR 6). (**b**) The IBD region containing the causative locus is located on OAR 6 between 109 and 115 Mb. (**c**) The black boxes show 35 cow reference genes encompassing the sheep IBD region based on the comparative genomic maps. (**d**) **&** (**e**) The boxes represent exons of the cow and the predicted sheep *DMP1* genes. The solid parts of boxes demonstrate the coding region for *DMP1*. The vertical lines on exon 6 are representing the mutant SNP (R145X) with the other 8 SNPs. (**f**) The number and color in each diamond box shows r^2^ value for each pair of SNPs. Higher numbers and darker colors indicate SNPs are with higher LD. Four SNPs in block 1 show a complete mutual linkage disequilibrium (LD) but not with the causative mutation (R145X).

The genotyping data using a SNP BeadChip for this study was submitted to the NCBI Gene Expression Omnibus (http://www.ncbi.nlm.nih.gov/geo) under Super Series accession no GSE26310. In our study, exactly 3 homozygous regions of more than 10 consecutive SNPs were identified in all 17 affected sheep whereas the 3 carriers exhibited heterozygosity in those regions. The largest homozygous segment consisted of 125 consecutive SNP loci, whereas the other two segments had only 19 or 11 consecutive SNPs. The largest 125-SNP region started from SNP OAR6_109334543.1 and extended to SNP s03175.1, covering a region of 5.95 Mb (109,334,543 to 115,285,275 bp) on the long arm of sheep chromosome 6 (OAR 6). There were 35 genes located in this region based on the bovine reference sequence (**Figures 2a, 2b** **and Table S2**). The size of the second homozygous segment was about 0.70 Mb (OAR6: 118,884,834 to 119,587,883 bp) and had only 3.2 Mb separation from the first homozygous segment on the same chromosome (OAR 6). The third homozygous segment covered a 0.66 Mb region (OAR15: 1,131,166 to 1,654,496 bp) on the short arm of OAR 15. Within the second and third homozygous regions there were 8 and 6 positional candidate genes, respectively (**Table S2**). One gene called *DMP1* in the largest 125-SNP region was considered the most plausible candidate due to its biological functions involved in mineralization and phosphate homeostasis and previous reports of its involvement in human rickets [8], [21]. By comparing with the bovine genomic reference sequence it was found that this gene was at 112.20 Mb on OAR6q (**Table S2**), spanned over 16 kb of size and contained 6 exons.

### Candidate Gene Sequencing

Fine mapping was carried out by individual PCR amplification and sequencing of all the exons of the *DMP1* gene to identify the mutation for this disease **(Table S1)**. After blasting the sheep genomic reference sequence with bovine *DMP1* coding sequence (ENSBTAT00000025468), there were several alignments for exon 6 with high identity rates (91%–96%). As exon 6 is the last and also the largest exon covering 88% of the open reading frame of *DMP1*, it was prioritized for re-sequencing. Direct sequencing from 3 carriers and 1 normal control revealed 1 published SNP (s18093.1, OAR6: 112,213,812 bp) and 8 novel ones in this exon (**Figure 2e**). The complete genomic sequence of exon 6 for sheep *DMP1* gene was submitted to GenBank (http://www.ncbi.nlm.nih.gov/genbank/) under accession no. HQ600592.

Moreover, we successfully amplified exon 1, exon 3, exon 4 and exon 5 of sheep *DMP1* gene using exon flanked intronic sequences of cow reference for each exon. The amplifications of exon 2 failed after using several pairs of primers. Intronic SNPs (SNP1 and SNP2) and a 1-bp insertion/deletion (indel: A) were identified respectively in intron 1, intron 5 and intron 3 by direct sequencing (**Table S1**).

### Mutation Analysis

**Figure 3:**
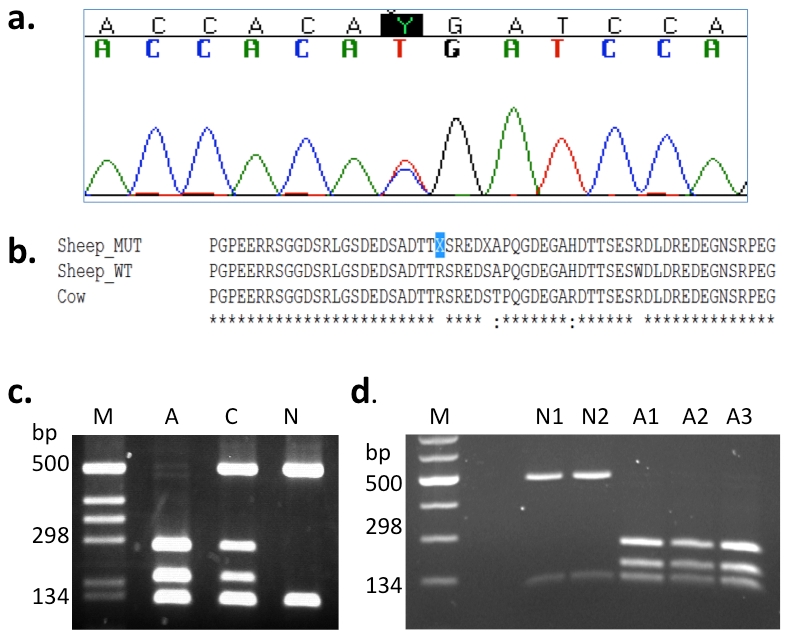
Mutation analyses. (**a**) The chromatogram shows the location of the mutation (Y: C/T). (**b**) Protein sequence alignments of DMP1. The highlighted R145X substitution induces a stop codon that leads to premature termination of the protein. MUT, R145X mutant; WT, wild type. (**c**) PCR-RFLP genotyping results for the affected (3 bands) and carrier (4 bands) Corriedale sheep and normal (2 bands) control sheep. M, 1kb marker; A, affected; C, carrier; N, normal. (**d**) RT-PCR-RFLP results for 2 controls (N1 &N2) and 3 affected sheep (A1, A2 &A3).

Seventeen base pairs upstream of SNP s18093.1, a SNP (C/T, OAR6:112,213,795 bp) located at +250bp of exon 6 caused a non-synonymous amino acid change in the DMP1 protein. This C -> T transition introduced a stop codon (R145X) that led to a truncated DMP1 protein at the 145^th^ amino acid (**Figure 3a** **,** **Figure 3b** and **Figure S1**). Genotyping of this mutation by PCR-RFLP (Polymerase Chain Reaction-Restriction Fragment Length Polymorphism) (**Figure 3c**) and direct sequencing showed all 17 affected animals had the “T T” genotype; the 3 carriers were “C T”; 24 phenotypically normal related sheep were either “C T” or “C C”; and 46 unrelated control sheep of other breeds were only “C C” genotypes (**Table 1**). This mutation was 100% concordant with the recessive pattern of inheritance in affected, carrier and normal individuals. Moreover, genotyping of the other 8 SNPs in exon 6 which surrounded the R145X mutation in *DMP1*, found incomplete linkage disequilibrium for these markers with either the disease or the mutant SNP (**Figure 2f**). The 3 genomic variants in introns 1, 3 and 5 identified were not located at either the splicing receptor or acceptor sites. Therefore, those mutations could be ruled out as candidate mutation loci. RT-PCR/RFLP (**Figure 3d**) was performed for the R145X mutation and showed the same digestion pattern as PCR-RFLP.

**Table 1:** The genotyping results of the causative mutation (R145X).

Sheep Breed	Phenotype		Genotype		Rate of Concordance
	Rickets Status	# Sheep	Genotype	# Sheep	
Corriedale	Affected	17	T T	17	100%
	Known Carrier	3	T C	3	100%
	Phenotype Normal^a^	24	T C , C C	24	100%
Texel, Perendale	Normal	46	C C	46	100%

a These sheep are phenotypically normal offspring from F1 generation and F2 backcross generations generated by out-crossing and back-crossing trials.

Taken together, our results strongly suggest that the R145X substitution is causative for inherited rickets in Corriedale sheep, disrupting the function of the DMP1 protein, preventing successful bone mineralization and phosphate homeostasis.

## Discussion

Homozygosity mapping is a powerful method to map simple recessive traits since the regions immediately flanking the mutation locus will likely be homozygous by descent in closely related offspring. The affected animals in this study resulted from consanguineous mating between pairs of offspring descended from one common male ancestor in the embryo transfer and backcross studies used to characterize the inheritance of the disease [15], [16]. The region containing the mutation should in these circumstances span a large chromosome segment that would be progressively diminished in subsequent meioses when recombination events occur near to the mutation. This mapping strategy has been used successfully for recessive diseases in both animal and human studies [20], [22]. The homozygosity mapping in this study was conducted from knowledge of the SNP genotypes in affected individuals and their genomic location, and used command line UNIX tools including awk, sort and join to identify any regions of at least 10 consecutive homozygous SNPs, equivalent to about 0.5 cM or more. It was presumed that one such region would contain the mutation associated with inherited rickets in Corriedale sheep. In this study, 3 homozygous regions met the criteria of exceeding 10 consecutive homozygous SNP markers. Previously reported rickets genes including *PHEX*, *FGF23*, *ENPP1*, *SLC34A3, CYP27B1* and *VDR* did not show up in any of these homozygous segments based on the ovine genome assembly v1.0, whereas *DMP1* was contained in the largest region. This is not surprising because *PHEX* or *FGF23* related rickets have a distinctive inheritance mode that differs from the autosomal recessive inheritance of the rickets studied here. The severe hypophosphatemia in affected sheep indicates disturbed phosphate balance within the body. Therefore, the *DMP1* gene was considered to be a high priority candidate gene. The nonsense mutation we identified in the *DMP1* gene was 100% concordant with the recessive pattern of inheritance for the disease studied.

*DMP1* encodes a serine-rich acidic protein and is a member of Small Integrin-Binding Ligand, N-linked, Glycoprotein family (SIBLING) which mineralizes tissue such as bones and teeth by binding strongly to hydroxyapatite using an Arg-Gly-Asp (RGD) tripeptide [23]. Dual functions of the DMP1 protein have been reported based on the *in vitro* studies. The nonphosphorylated cytoplasmic DMP1 protein translocates to the cell nucleus and initially acts as a transcriptional factor to enhance gene transcription of the osteoblast-specific genes such as alkaline phosphatase and osteocalcin, and then it moves out to the extracellular matrix during the osteoblast to osteocyte transition phase, to promote mineralization and phosphate homeostasis [24], [25]. Loss of function mutations in the human *DMP1* gene have been shown to cause autosomal recessive hypophosphatemic rickets in different ethnic groups including Turkish, consanguineous Spanish, Lebanese and Japanese populations. These variant mutations in *DMP1* include deletions in exon 6, nucleotide substitution in the splice acceptor sequence of intron 2, and missense mutations in exon 2 or exon 3 that introduce the premature termination codon [8], [21], [26]. The *Dmp1*-null mouse model also exhibits hypophosphatemic rickets, which indicates the important role of the *DMP1* gene in the development of normal bone formation [21]. Since the majority of the dentin matrix protein 1 is encoded by the sixth and last exon of *DMP1*, typical for proteins in the SIBLING family [27], the novel nonsense mutation (250C/T, R145X) identified here in ovine *DMP1* gene is a plausible mutation leading to loss of function following nonsense-mediatedmRNA decay shown to occur in human patients. The nonsense-mediated mRNA decay is a post-transcriptional mechanism in eukaryotic species. It likely eliminates the abnormal transcripts produced by prematurely terminated translation [28]. The previous studies strongly support the nonsense mutation we identified in exon 6 as causing loss of function of the DMP1 protein and being responsible for ARHR type 1 in Corriedale sheep.

Despite the wide variety of mutated genes that result in the different forms of hereditary hypophosphatemic rickets, the clinical features are relatively similar (with the exception of HHRH). In XLH, ADHR, ARHR type 1 and 2, the overriding common feature is increased serum concentration and activity of FGF23. FGF23 inhibits a NPT-2a co-transporter (Na-P_i_ co-transporter) in the kidneys leading to loss of phosphate from renal tubules into the urine (phosphaturia). FGF23 also inhibits the activity of CYP27B1, thereby inhibiting active vitamin D (1,25-dihydroxyvitamin D_3_) production [29]. As a result of the increased FGF23 concentrations patients with XLH, ADHR, ARHR type 1 and 2 present with rickets, hypophosphatemia, phosphaturia and inappropriately normal serum 1,25-dihydroxyvitamin D_3_ concentrations [9], [30]. Corriedale sheep with the mutation in *DMP1* also developed rickets, were severely hypophosphataemic and had normal serum 1,25(OH)_2_D_3_ concentrations [1], [16]. The mechanism by which mutations in *PHEX*, *DMP1* or *ENPP1* result in upregulation of FGF23 is unknown. An unusual feature of ARHR type 2 is that mutations in *ENPP1* are also associated with generalized arterial calcification of infancy, again the mechanism by which a mutation in *ENPP1* can result in a disease characterised by hypomineralization and one characterised by hypermineralization is unknown [9]. In HHRH, CYP27B1 is actually upregulated, resulting in increased 1,25(OH)_2_D_3_ concentrations, and perhaps explaining the hypercalciuria [11].

In conclusion, this is the first report of ARHR type 1 caused by a mutation in the *DMP1* gene in a sheep population. In this study, we demonstrate successful use of applying high density SNP arrays for localization of a mutation locus causing a recessive inherited defect. The results can be rapidly applied as a selection marker to identify carriers with the defective “T” allele in order to avoid at-risk matings to improve animal welfare and decrease economic losses. At this stage, the incidence of the mutation in the Corriedale sheep population is unknown, however it is suspected to be widespread and testing of New Zealand Corriedale sheep is planned. More importantly, due to their size and relatively low cost of maintenance, Corriedale sheep with inherited rickets could be used as an animal model for this form of human rickets. Even though the human and sheep mutations are not exactly the same, both disrupt the function of the DMP1 protein. The sheep model can be used for further investigation of the role of DMP1 in phosphate homeostasis and to explore the potential for early medical intervention to reduce the development of the disease in homozygous individuals.

## Materials and Methods

### Ethics statement

Approvals dealing with DNA or tissue collection from animals were obtained from the Massey University Animal Ethics Committee (APPROVAL NUMBER 05/123) and Iowa State University Animal Care & Use Committee did not require additional approvals.

### Animals

In total, 17 affected Corriedale sheep (9 males, 8 females) and 27 phenotypically normal crossbred Corriedale sheep (6 males, 21 females) as well as 46 normal controls (Texel and crossbred Perendale sheep) were examined. The age of examined animals ranged from a fetus of 133 days gestation to a 3 year-old animal. Eight affected sheep were from the original commercial property at Marlborough, New Zealand, and 9 affected sheep were their offspring. The 27 phenotypically normal crossbred Corriedale sheep were progeny from the mating of unrelated Romney ewes with the Corriedale carrier ram (F1 generation from outcross), or progeny of the F1 generation daughters mated back to the carrier ram (F2 generation from backcross). SNP genotypes were obtained for the 17 affected and 3 carrier Corriedale sheep, while fine mapping was conducted on all 44 pure or crossbred Corriedale sheep. The 46 normal control sheep used for validation of the mutation were from unrelated populations without evidence of rickets.

### SNP genotyping

DNA was extracted from blood using a Roche MagNA Pure automated analyser. DNA samples were randomized on genotyping plates according to disease status. The genotyping was performed using the Ovine SNP50 BeadChip containing 54,241 SNPs (Illumina, San Diego, CA, USA) with standard procedures at GeneSeek Inc. Lincoln, NE, USA. The SNPs retained had to get a call rate >80% and a minimum GenTrain score of 0.25. All genotypes with satisfactory call rate and GenTrain score were used regardless of minor allele frequency (MAF).

### SNP chip data analyses

The IBD program used awk, join and sort UNIX commands to scan the whole genome of the genotyped affected cases, to produce a list of all the strings of consensus homozygous markers with a length of more than 10 SNPs. The same process was repeated in the carriers to ensure the regions identified in the affected individuals were not homozygous in the carriers. The homozygosity mapping algorithm involved the following: first, SNPs in the Illumina ovine 50K map file were sorted by chromosome and position, and numerically coded in contiguous order. Second, the names of the “A/B” genotypes (provided by GeneSeek Inc.) for each individual were joined with the renumbered map file to identify the genotypes by their locations. The joined file was separated into three groups according to the disease status of affected, carrier and normal. Third, the number of occurrences of genotypes (“AA”, “AB”, “BB” and no call “—”) for each SNP was counted in the affected and carrier groups. Fourth, the genotype frequencies were calculated for each SNP. In the frequency calculation, the numerator was the count of genotypes being either “AA”, “AB” or “BB”, and the denominator was the total count excluding the category of no call. Finally, if the highest genotype frequency for a SNP was equal to 1 and the two alleles of this genotype were the same, such as “AA” or “BB”, this SNP was considered a homozygous locus. The span of contiguous homozygous loci were accumulated and reported if the region included more than 10 loci. Genes in these IBD regions were examined for potential involvement using comparative maps between the ovine and bovine genomes through the Ovine Genome Assembly v1.0 (http://www.livestockgenomics.csiro.au/perl/gbrowse.cgi/oar1.0/#search).

### *DMP1* gene sequencing

Primers were designed to amplify all 6 exons for sheep *DMP1* (**Table S1**). Cow *DMP1* genomic sequence was used as a template for primer designing in order to amplify the first 5 exons of sheep *DMP1* gene. PCR was performed using a 10 µl system with GoTaq DNA polymerase (Promega, Madison, WI). The annealing temperatures were 58°C or 60°C. PCR products were purified with ExoSAP-IT® (USB, Cleveland, OH), before pooling from 3 carriers and sequencing commercially using a 3730xl DNA Analyzer.

### Mutation analyses

Sheep EST sequence (DY509762) encompassing exon 1 to exon 5 of sheep *DMP1* along with re-sequencing results were used to predict the DMP1 protein sequence by applying the ExPASy translate tool (http://ca.expasy.org/tools/dna.html). The predicted sheep DMP1 protein was compared to the wild type (normal) of sheep and cow (AAI49017) using the CLUSTALW alignment tool (http://align.genome.jp/). Each SNP variant was analyzed to check whether it could induce a missense or nonsense mutation. The identified SNPs were genotyped on all 44 related Corriedale sheep and/or 46 normal controls by restriction enzyme digestion (PCR-RFLP) or direct sequencing. The genotyping results from all 9 SNPs distributed in exon 6 of the *DMP1* gene were formatted and put into Haploview software (http://www.broadinstitute.org/haploview/haploview). The linkage disequilibrium (LD) between pairs of SNPs in exon 6 was analyzed by Haploview software. Under the LD tabs, r^2^, the correlation coefficient, was saved to show the LD between each pair of SNPs. The related LD color scheme was produced accordingly.

### RNA extraction and Reverse transcriptase-polymerase chain reaction (RT-PCR)

RNA was extracted from fibroblast cells cultured *in vitro* from skin biopsies of affected and control sheep to confirm the mutation existed at the transcript level. The fibroblast cell culture is as previously described [16]. Total RNA was extracted using Tri Reagent as per the manufacturer's instructions (Sigma-Aldrich Co., USA). RT-PCR was performed using the Superscript® one-step RT-PCR system with Platinum® Taq polymerase (Invitrogen Corp., USA). The RT-PCR conditions were: 55°C for 30 min, 94°C for 2 min, 40 cycles of 30 s at 94°C, 30 s at 58° and 30 s at 72°C, and finally a 5 min elongation step at 72°C.

### Restriction enzyme digestion

The nonsense mutation (C -> T) created a new recognition site for the restriction enzyme NIaIII (New England Biolabs Inc., USA). DNA fragments from the PCR and RT-PCR reactions were subjected to restriction enzyme digestion with NlaIII. The reaction mix was incubated at 37°C overnight, and the products were analyzed on a 2.5% (w/v) ultra-pure agarose gel.

## Supporting Information

Figure S1 **Complete predicted DMP1 protein sequence alignments among rickets affected sheep, wild type sheep and cow.** The amino acid (R145X) with a border is the position where the “C - > T” transition induced a stop codon and lead to a truncated DMP1 protein at the 145^th^ amino acid.
(TIF)

Table S1 **Primer sequence, annealing temperature, PCR amplicon information and genetic variants identified by sequencing.**
(DOC)

Table S2 **The list of ovine positional candidate genes based on the bovine reference gene sequences.**
(DOC)
