# Supplementary figures and images for: A Novel Nonsense Mutation in the DMP1 Gene Identified by a Genome-Wide Association Study Is Responsible for Inherited Rickets in Corriedale Sheep
Source: PLoS One. 2011 Jul 1;6(7):e21739. doi: 10.1371/journal.pone.0021739 (PMC3128599; doi:10.1371/journal.pone.0021739)

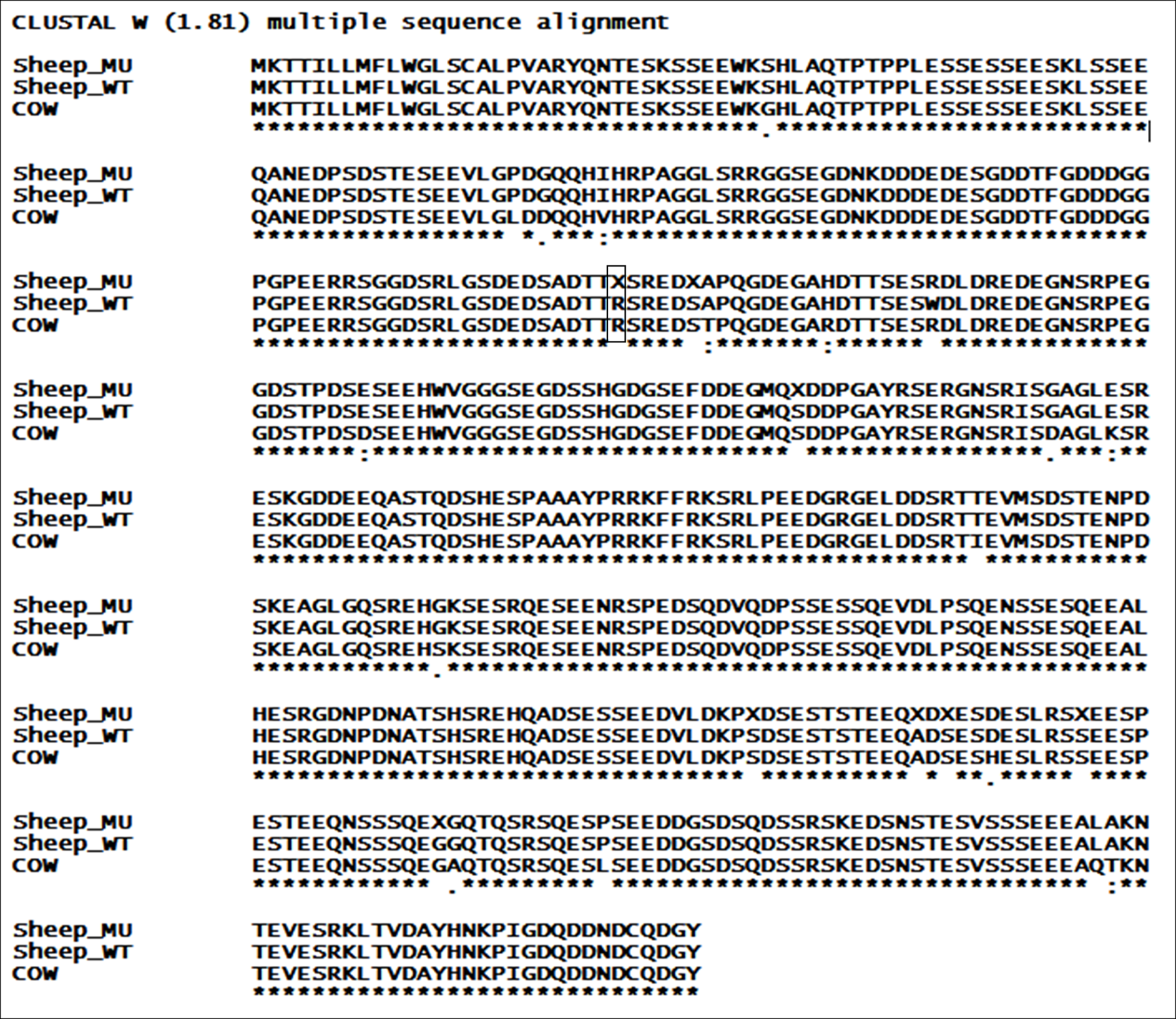

Supplement: Figure S1 — Complete predicted DMP1 protein sequence alignments among rickets affected sheep, wild type sheep and cow. The amino acid (R145X) with a border is the position where the “C - > T” transition induced a stop codon and lead to a truncated DMP1 protein at the 145th amino acid. (TIF) [file pone.0021739.s001.tif]
