# Supplementary material for: A Novel Nonsense Mutation in the DMP1 Gene Identified by a Genome-Wide Association Study Is Responsible for Inherited Rickets in Corriedale Sheep
Source: PLoS One. 2011 Jul 1;6(7):e21739. doi: 10.1371/journal.pone.0021739 (PMC3128599; doi:10.1371/journal.pone.0021739)
Supplement: Table S1 — Primer sequence, annealing temperature, PCR amplicon information and genetic variants identified by sequencing. (DOC) [file pone.0021739.s002.doc]

Table S1. Primer sequence, annealing temperature, PCR amplicon information and genetic variants identified by sequencing.

| **Primer name** | **Primer sequence** | **Anealing Temp. (ºC)** | **Size (bp)** | **Fragment name** | **Genetic variant (position in the fragment)** | **SNP name** | **SNP characteristic** |
| --- | --- | --- | --- | --- | --- | --- | --- |
| DMP1e1F | 5’- CTTTTCCCATCCTTGGGTCT-3’ | 60 | 560 | DMP1e1 | C/T (+ 453bp) | SNP1 | intronic |
| DMP1e1R | 5’- GCACTGTTCTCCCCATCTTT-3’ |  |  |  |  |  |  |
| DMP1e3F | 5’- CAAAATGTTATCCCCAGACCA-3’ | 60 | 388 | DMP1e3 | no | - | no |
| DMP1e3R | 5’- TTCAGCATTTGCAGTGAAGC-3’ |  |  |  |  |  |  |
| DMP1e4F | 5’- GGCTAAACACAAGCCAAGGA-3’ | 60 | 659 | DMP1e4 | indel (A) (+284bp) | - | intronic |
| DMP1e4R | 5’- GGAGATTGGGAGGGTCATGT-3’ |  |  |  |  |  |  |
| DMP1e5F | 5’- CAGGCCATTTGGAAAGTCAT-3’ | 60 | 411 | DMP1e5 | C/G (+ 341bp) | SNP2 | intronic |
| DMP1e5R | 5’- GAGGAACATTTAGGGCCACA-3’ |  |  |  |  |  |  |
| DMP1e6_2F ^a^  DMP1e6_2R^a^ | 5’- ATGGAAAATGGGGTGACTTG-3’  5’- CATCCCTTCATCGTCGAACT-3’ | 58 | 668 | DMP1e6_2 | C/T (+455bp)  C/T (+472bp)  A/G (+493bp) | R145X  S18033.1  SNP3 | exonic  exonic  exonic |
| DMP1e6_5F  DMP1e6_5R | 5’- ATGAGTCCAGGGGTGACAAC-3’  5’- CAACAATGGGCATCTTTCCT-3’ | 58 | 703 | DMP1e6_5 | C/G (+101bp)  C/T (+134bp)  C/T (+140bp)  A/G (+167bp)  A/C (+218bp)  C/T (+602bp) | SNP4  SNP5  SNP6  SNP7  SNP8  SNP9 | exonic  exonic  exonic  exonic  exonic  3’ UTR |

^a^ These primer sets were used for PCR-RFLP genotyping of the mutation R145X
