# Supplementary material for: A Novel Nonsense Mutation in the DMP1 Gene Identified by a Genome-Wide Association Study Is Responsible for Inherited Rickets in Corriedale Sheep
Source: PLoS One. 2011 Jul 1;6(7):e21739. doi: 10.1371/journal.pone.0021739 (PMC3128599; doi:10.1371/journal.pone.0021739)
Supplement: Table S2 — The list of ovine positional candidate genes based on the bovine reference gene sequences. (DOC) [file pone.0021739.s003.doc]

Table S2 The list of ovine positional candidate genes based on the bovine reference gene sequences

| **Targeted Region (size)** | **Ovine Chromosome Position** | **Bovine Ref. Gene Symbol** | **Bovine Ref. Gene Name** | **Bovine Gene Accession No.** |
| --- | --- | --- | --- | --- |
| 1 | OAR6:109103268..109364732 | WDFY3 | Similar to WD repeat and FYVE domain-containing protein 3 (Autophagy-linked FYVE protein) (Alfy) | [XM_617252](http://www.ncbi.nlm.nih.gov/entrez/query.fcgi?db=nucleotide&cmd=search&term=XM_617252) |
| (5.95 Mb) | OAR6:110461394..110535578 | ARHGAP24 | Rho GTPase activating protein 24 | [NM_001102234](http://www.ncbi.nlm.nih.gov/entrez/query.fcgi?db=nucleotide&cmd=search&term=NM_001102234) |
|  | OAR6:111226629..111449410 | PTPN13 | Protein tyrosine phosphatase, non-receptor type 13 (APO-1/CD95 (Fas)-associated phosphatase) | [NM_174590](http://www.ncbi.nlm.nih.gov/entrez/query.fcgi?db=nucleotide&cmd=search&term=NM_174590) |
|  | OAR6:111458495..111488852 | SLC10A6 | Solute carrier family 10 (sodium/bile acid cotransporter family), member 6 | [NM_001081738](http://www.ncbi.nlm.nih.gov/entrez/query.fcgi?db=nucleotide&cmd=search&term=NM_001081738) |
|  | OAR6:111512006..111515283 | UBE2D3P | Similar to Putative ubiquitin-conjugating enzyme E2 D3-like protein | [NM_001075135](http://www.ncbi.nlm.nih.gov/entrez/query.fcgi?db=nucleotide&cmd=search&term=NM_001075135) |
|  | OAR6:111512014..111515294 | LOC783264 | Similar to ubiquitin-conjugating enzyme E2D 3 | [XM_001249763](http://www.ncbi.nlm.nih.gov/entrez/query.fcgi?db=nucleotide&cmd=search&term=XM_001249763) |
|  | OAR6:111512014..111515294 | LOC783114 | Similar to ubiquitin-conjugating enzyme E2D 3 | [XM_001250124](http://www.ncbi.nlm.nih.gov/entrez/query.fcgi?db=nucleotide&cmd=search&term=XM_001250124) |
|  | OAR6:111600842..111740902 | AFF1 | Similar to AF4/FMR2 family, member 1 | [XM_001249488](http://www.ncbi.nlm.nih.gov/entrez/query.fcgi?db=nucleotide&cmd=search&term=XM_001249488) |
|  | OAR6:111756587..111811197 | KLHL8 | Similar to KIAA1378 protein, transcript variant 1 | [XM_612186](http://www.ncbi.nlm.nih.gov/entrez/query.fcgi?db=nucleotide&cmd=search&term=XM_612186) |
|  | OAR6:111885205..111904838 | HSD17B13 | Hydroxysteroid (17-beta) dehydrogenase 13 | [NM_001046616](http://www.ncbi.nlm.nih.gov/entrez/query.fcgi?db=nucleotide&cmd=search&term=NM_001046616) |
|  | OAR6:111920170..111969527 | HSD17B11 | Hydroxysteroid (17-beta) dehydrogenase 11 | [NM_001046286](http://www.ncbi.nlm.nih.gov/entrez/query.fcgi?db=nucleotide&cmd=search&term=NM_001046286) |
|  | OAR6:112013056..112050582 | NUDT9 | Nudix (nucleoside diphosphate linked moiety X)-type motif 9 | [NM_001101096](http://www.ncbi.nlm.nih.gov/entrez/query.fcgi?db=nucleotide&cmd=search&term=NM_001101096) |
|  | OAR6:112063439..112116046 | SPARCL1 | SPARC-like 1 (hevin) | [NM_001034302](http://www.ncbi.nlm.nih.gov/entrez/query.fcgi?db=nucleotide&cmd=search&term=NM_001034302) |
|  | **OAR6:112199546..112216300*** | **DMP1*** | **Dentin matrix acidic phosphoprotein 1** | [**NM_174038**](http://www.ncbi.nlm.nih.gov/entrez/query.fcgi?db=nucleotide&cmd=search&term=NM_174038) |
|  | OAR6:112365289..112411784 | MAN2B2 | Similar to mannosidase, alpha, class 2B, member 2 | [XM_601803](http://www.ncbi.nlm.nih.gov/entrez/query.fcgi?db=nucleotide&cmd=search&term=XM_601803) |
|  | OAR6:112588875..112650749 | PPP2R2C | Similar to Serine/threonine-protein phosphatase 2A 55 kDa regulatory subunit B gamma isoform | [XM_001250700](http://www.ncbi.nlm.nih.gov/entrez/query.fcgi?db=nucleotide&cmd=search&term=XM_001250700) |
|  | OAR6:112757862..112757903 | LOC617028 | Hypothetical LOC617028 | [XM_882441](http://www.ncbi.nlm.nih.gov/entrez/query.fcgi?db=nucleotide&cmd=search&term=XM_882441) |
|  | OAR6:112759544..112781041 | LOC782334 | Hypothetical LOC782334 | [XM_001250977](http://www.ncbi.nlm.nih.gov/entrez/query.fcgi?db=nucleotide&cmd=search&term=XM_001250977) |
|  | OAR6:112780837..112910375 | JAKMIP1 | Janus kinase and microtubule interacting protein 1 | [NM_001102251](http://www.ncbi.nlm.nih.gov/entrez/query.fcgi?db=nucleotide&cmd=search&term=NM_001102251) |
|  | OAR6:113024908..113058856 | LOC615433 | Hypothetical LOC615433 | [XM_867237](http://www.ncbi.nlm.nih.gov/entrez/query.fcgi?db=nucleotide&cmd=search&term=XM_867237) |
|  | OAR6:113144679..113208484 | CRMP1 | Similar to Dihydropyrimidinase-related protein 1 (DRP-1) (Collapsin response mediator protein 1) | [XM_580336](http://www.ncbi.nlm.nih.gov/entrez/query.fcgi?db=nucleotide&cmd=search&term=XM_580336) |
|  | OAR6:113213443..113312073 | EVC | Ellis van Creveld syndrome | [NM_174747](http://www.ncbi.nlm.nih.gov/entrez/query.fcgi?db=nucleotide&cmd=search&term=NM_174747) |
|  | OAR6:113335352..113473188 | EVC2 | Ellis van Creveld syndrome 2 | [NM_173927](http://www.ncbi.nlm.nih.gov/entrez/query.fcgi?db=nucleotide&cmd=search&term=NM_173927) |
|  | OAR6:113451908..113473188 | LOC786485 | Hypothetical LOC786485 | [XM_001254147](http://www.ncbi.nlm.nih.gov/entrez/query.fcgi?db=nucleotide&cmd=search&term=XM_001254147) |
|  | OAR6:113787927..114038860 | STK32B | Similar to serine/threonine kinase 32B | [XM_607574](http://www.ncbi.nlm.nih.gov/entrez/query.fcgi?db=nucleotide&cmd=search&term=XM_607574) |
|  | OAR6:114257470..114261815 | CYTL1 | Similar to Cytokine-like protein 1 precursor (Protein C17) | [XM_581416](http://www.ncbi.nlm.nih.gov/entrez/query.fcgi?db=nucleotide&cmd=search&term=XM_581416) |
|  | OAR6:114391388..114395645 | MSX1 | Msh homeobox 1 | [NM_174798](http://www.ncbi.nlm.nih.gov/entrez/query.fcgi?db=nucleotide&cmd=search&term=NM_174798) |
|  | OAR6:114700887..114900429 | STX18 | Syntaxin 18 | [NM_001099719](http://www.ncbi.nlm.nih.gov/entrez/query.fcgi?db=nucleotide&cmd=search&term=NM_001099719) |
|  | OAR6:114899716..114940430 | NSG1 | Neuron specific gene family member 1 | [NM_001077957](http://www.ncbi.nlm.nih.gov/entrez/query.fcgi?db=nucleotide&cmd=search&term=NM_001077957) |
|  | OAR6:114899716..114901567 | LOC789276 | Hypothetical LOC789276 | [XM_001256071](http://www.ncbi.nlm.nih.gov/entrez/query.fcgi?db=nucleotide&cmd=search&term=XM_001256071) |
|  | OAR6:115074361..115095979 | ZNF509 | Similar to hCG2039195, transcript variant 1 | [XM_583985](http://www.ncbi.nlm.nih.gov/entrez/query.fcgi?db=nucleotide&cmd=search&term=XM_583985) |
|  | OAR6:115101064..115121797 | LYAR | Ly1 antibody reactive homolog (mouse) | [NM_001031769](http://www.ncbi.nlm.nih.gov/entrez/query.fcgi?db=nucleotide&cmd=search&term=NM_001031769) |
|  | OAR6:115135963..115151278 | TMEM128 | Transmembrane protein 128 | [NM_001034454](http://www.ncbi.nlm.nih.gov/entrez/query.fcgi?db=nucleotide&cmd=search&term=NM_001034454) |
|  | OAR6:115207964..115221470 | LOC525643 | Similar to otopetrin | [XM_603996](http://www.ncbi.nlm.nih.gov/entrez/query.fcgi?db=nucleotide&cmd=search&term=XM_603996) |
|  | OAR6:115249664..115251638 | DRD5 | Dopamine receptor D5 | [XM_604584](http://www.ncbi.nlm.nih.gov/entrez/query.fcgi?db=nucleotide&cmd=search&term=XM_604584) |
| 2 | OAR6:118851890..118883051 | PDE6B | Phosphodiesterase 6B, cGMP-specific, rod, beta | [NM_174418](http://www.ncbi.nlm.nih.gov/entrez/query.fcgi?db=nucleotide&cmd=search&term=NM_174418) |
| (0.70 Mb) | OAR6:118941179..118943474 | GPI7 | GPI7 protein | [NM_001024518](http://www.ncbi.nlm.nih.gov/entrez/query.fcgi?db=nucleotide&cmd=search&term=NM_001024518) |
|  | OAR6:118976061..119063813 | LOC614799 | Similar to solute carrier family 2 (facilitated glucose transporter), member 9 | [XM_866430](http://www.ncbi.nlm.nih.gov/entrez/query.fcgi?db=nucleotide&cmd=search&term=XM_866430) |
|  | OAR6:119204026..119245137 | WDR1 | WD repeat domain 1 (WDR1) | [NM_001046346](http://www.ncbi.nlm.nih.gov/entrez/query.fcgi?db=nucleotide&cmd=search&term=XM_866430) |
|  | OAR6:119417009..119417107 | LOC786062 | Similar to Zinc finger protein 518B-like | [XM_001788422](http://www.ncbi.nlm.nih.gov/entrez/query.fcgi?db=nucleotide&cmd=search&term=XM_001788422) |
|  | OAR6:119417009..119417587 | ZNF518B | Similar to Zinc finger protein 518B | [XM_584181](http://www.ncbi.nlm.nih.gov/entrez/query.fcgi?db=nucleotide&cmd=search&term=XM_584181) |
| 3 | OAR15:1220402..1223574 | KIAA1826 | Hypothetical protein LOC511226 | [NM_001046078](http://www.ncbi.nlm.nih.gov/entrez/query.fcgi?db=nucleotide&cmd=search&term=NM_001046078) |
| (0.66 Mb) | OAR15:1338164..1344393 | KBTBD3 | Kelch repeat and BTB (POZ) domain containing 3 | [XM_602528](http://www.ncbi.nlm.nih.gov/entrez/query.fcgi?db=nucleotide&cmd=search&term=XM_602528) |
|  | OAR15:1418829..1442937 | AASDHPPT | Similar to aminoadipate-semialdehyde dehydrogenase-phosphopantetheinyl transferase | [XM_001250765](http://www.ncbi.nlm.nih.gov/entrez/query.fcgi?db=nucleotide&cmd=search&term=XM_001250765) |
|  | OAR15:1484534..1488246 | ANKRD49 | Ankyrin repeat domain 49 | [NM_001014965](http://www.ncbi.nlm.nih.gov/entrez/query.fcgi?db=nucleotide&cmd=search&term=NM_001014965) |
|  | OAR15:1503540..1575517 | MRE11A | Double-strand break repair protein meiotic recombination 11 homolog A | [XM_603439](http://www.ncbi.nlm.nih.gov/entrez/query.fcgi?db=nucleotide&cmd=search&term=XM_603439) |
|  | OAR15:1596294..1619791 | GPR83 | Similar to G protein-coupled receptor 83 | [XM_615580](http://www.ncbi.nlm.nih.gov/entrez/query.fcgi?db=nucleotide&cmd=search&term=XM_615580) |
|  | OAR15:1220402..1223574 | KIAA1826 | Hypothetical protein LOC511226 | [NM_001046078](http://www.ncbi.nlm.nih.gov/entrez/query.fcgi?db=nucleotide&cmd=search&term=NM_001046078) |

*The most plausible candidate gene for inherited rickets in Corriedale sheep.
